# A brain tumor segmentation enhancement in MRI images using U-Net and transfer learning

**DOI:** 10.1186/s12880-025-01837-4

**Published:** 2025-07-31

**Authors:** Amin Pourmahboubi, Nazanin Arsalani Saeed, Hamed Tabrizchi

**Affiliations:** 1 Department of Computer Science, Faculty of Mathematics, Statistics, and Computer Science, University of Tabriz, Tabriz, East Azerbaijan Iran; 2 Department of Biology, Faculty of Natural Science, University of Tabriz, Tabriz, East Azerbaijan Iran

**Keywords:** Brain tumor segmentation, Convolutional neural networks (CNNs), Deep learning, Magnetic resonance imaging (MRI), Medical image analysis, Neuroimaging, Transfer learning, U-Net architecture

## Abstract

This paper presents a novel transfer learning approach for segmenting brain tumors in Magnetic Resonance Imaging (MRI) images. Using Fluid-Attenuated Inversion Recovery (FLAIR) abnormality segmentation masks and MRI scans from The Cancer Genome Atlas’s (TCGA’s) lower-grade glioma collection, our proposed approach uses a VGG19-based U-Net architecture with fixed pretrained weights. The experimental findings, which show an Area Under the Curve (AUC) of 0.9957, F1-Score of 0.9679, Dice Coefficient of 0.9679, Precision of 0.9541, Recall of 0.9821, and Intersection-over-Union (IoU) of 0.9378, show how effective the proposed framework is. According to these metrics, the VGG19-powered U-Net outperforms not only the conventional U-Net model but also other variants that were compared and used different pre-trained backbones in the U-Net encoder.

**Clinical trial registration**

Not applicable as this study utilized existing publicly available dataset and did not involve a clinical trial.

## Introduction

Brain tumors are one of the most serious types of neurological conditions, creating major challenges for healthcare systems around the world [1]. They can cause severe symptoms and are often linked to high rates of illness and death. Early and accurate diagnosis is vital for effective treatment and better patient outcomes. Magnetic Resonance Imaging (MRI) is the go-to method for detecting and analyzing brain tumors because it provides clear images of soft tissues and is non-invasive [2, 3]. Accurately segmenting brain tumors from MRI scans is a key part of the clinical process [4]. It helps doctors define tumor edges, track disease progression, and plan surgeries or treatments. While manual segmentation by expert radiologists is considered the best approach, it takes a lot of time and effort and can vary between different radiologists or even the same one at different times [5]. This has created a strong need for automated segmentation methods that can offer reliable and consistent results. Automating brain tumor segmentation is challenging because tumors are complex and vary widely. Tumors can differ in size, shape, location, and texture, and features like swelling, dead tissue, and contrast enhancement can make it harder to clearly define tumor edges, sometimes making them look like healthy tissue. Differences in MRI scan techniques and image artifacts can also negatively impact the accuracy of segmentation [6].

Traditional image processing techniques, like thresholding, region growing, and edge detection, have been used to tackle the brain tumor segmentation challenge [7]. While these methods can work well in certain cases, they often fall short when it comes to consistency and adaptability. They depend on handcrafted features and fixed parameters, making them sensitive to noise and variations in image quality [7]. These limitations make it clear that more advanced, flexible methods are needed. The rise of deep learning has transformed medical image analysis, providing powerful tools for automated feature extraction and pattern recognition. Convolutional Neural Networks (CNNs), in particular, have shown great success in various image analysis tasks because they can learn complex features directly from the data [8]. CNNs have been applied to brain tumor segmentation with impressive results, significantly outperforming traditional methods [3, 9].

Among the deep learning models transforming medical image analysis, U-Net has become a popular choice for biomedical image segmentation [10]. U-Net has a symmetrical encoder-decoder structure with skip connections that help combine both contextual and spatial information [11]. This allows the network to capture high-level semantic features and fine details, making it ideal for segmenting complex anatomical structures. Despite U-Net’s effectiveness, challenges remain when it comes to accurately segmenting brain tumors, particularly in areas with complex boundaries and varied regions [11, 12]. To improve U-Net’s performance, one strategy is to use pre-trained networks as the encoder backbone, taking advantage of the rich features learned from larges. VGG-19, a deep CNN known for its simplicity and depth, has been successfully used in many computer vision tasks and serves as a strong feature extractor [13]. It should be noted that VGG-19 has 19 layers with small convolutional filters, enabling it to capture detailed features at different scales [14]. By integrating VGG-19 into the U-Net architecture, the model can benefit from transfer learning, where knowledge from one task helps improve performance on another [15]. This is especially useful in medical imaging, where labelled data is often scarce due to the time and expertise required for manual annotation.

In this paper, we present a new brain tumor segmentation model that combines U-Net with VGG-19 as the encoder backbone. The goal is to improve U-Net’s feature extraction abilities by incorporating the deep, rich representations learned by VGG-19. By using pre-trained weights, we expect our model to better capture the complex features of tumors and speed up the training process. We evaluate the model on publicly available TCGA lower-grade glioma dataset [16, 17]. Our experiments involve a detailed comparison with the standard U-Net and other leading segmentation methods. We assess performance using metrics like the Dice Similarity Coefficient (DSC), Intersection Over Union (IOU) and overall segmentation accuracy.

The results show that our model outperforms others, especially when it comes to accurately outlining tumor boundaries and identifying varied tumor regions. The VGG-19 backbones improved feature extraction leads to better generalization across different patients and MRI conditions. We also explore how transfer learning influences the model’s performance.

### Motivation and contribution

The motivation for our proposed method comes from the need to improve the accuracy and reliability of automated brain tumor segmentation. As illusrated in recent extensive reviews such as [7, 18], they underscore the crucial importance of deep learning in attaining state-of-the-art performance in brain tumor segmentation, especially via CNN architectures such as U-Net. Our research advances these developments. A recent review of semi-automatic techniques for brain tumor segmentation and classification [19] highlights the increasing significance of deep learning in conjunction with pre-processing methods such as image registration and bias field correction, thereby strengthening the basis for our approach to enhance clinical applicability. While U-Net has proven effective in biomedical image segmentation, it might fall short when it comes to capturing the complex features of brain tumors, such as their irregular shapes, varying sizes, and diverse textures. By incorporating VGG-19 as the encoder backbone within the U-Net architecture, we aim to harness its deep feature extraction capabilities to tackle these challenges. This combination is expected to enhance the models ability to recognize detailed tumor characteristics, resulting in more accurate segmentation outcomes. It is undeniable that prior studies on brain tumor segmentation in MRI images have employed U-Net architectures or examined various pre-trained backbones; however, they have not thoroughly explored the integration of a VGG19-based U-Net with fixed pretrained weights in conjunction with the Focal Tversky loss function to enhance segmentation efficacy and also they typically rely on standard losses like binary cross-entropy or Dice loss. As mentioned in the manuscript, our experimentals and simulations led to employing Focal Tversky loss with parameters $\textrm{alpha}\,=\,0.7$ and $\textrm{gamma}\,=\,0.75$, resulting in a model that is specifically designed to address class imbalance and prioritize accurate segmentation. This is complemented by a distinctive training strategy, including an aggressive learning rate of 0.05 stabilized by batch normalization. In a nutshell, the innovation of this manuscript is expressed as follows: this manuscript presents a novel methodology using a VGG19-based U-Net architecture with the Focal Tversky loss function to attain superior brain tumor segmentation results on the TCGA lower-grade glioma dataset. Our proposed method efficiently extracts features from a pre-trained model, while the Focal Tversky loss function improves segmentation accuracy. This results in exceptional results, and exhibits distinct superiority over the traditional U-Net and other pre-trained backbone variations, marking an advancement in the field by establishing a high standard for segmentation accuracy in this subject.

The main challenge we aim to address is the difficulty of accurately segmenting brain tumors from MRI scans due to their complex and varied nature. Current methods, including standard U-Net models, often struggle to clearly define tumor boundaries. This gap in existing research underscores the need for a more advanced segmentation approach that can adapt to imaging conditions, delivering consistent and accurate results that are essential for informed clinical decision-making. The main contribution of this paper is to present a new brain tumor segmentation model that integrates VGG-19 as the encoder backbone within the U-Net architecture. The innovation of our approach comes from combining U-Net’s strong segmentation capabilities with VGG-19s advanced feature extraction, further enhanced by transfer learning from pre-trained weights. This combination allows the model to capture detailed and multi-level features of brain tumors, leading to improved segmentation accuracy, particularly in challenging cases. By addressing the research gap in this case, our method offers a more reliable and generalizable tool for automated brain tumor segmentation, which can support clinicians in diagnosis and treatment planning. In a nutshell, the main contributions of this paper are:

1. We propose a U-Net-based brain tumor segmentation model that incorporates VGG-19 as the encoder backbone, using pre-trained weights for enhanced feature representation.
2. We provide a comprehensive evaluation of the model on challenging brain tumor MRI datasets, showing substantial improvements compared to existing methods.
3. We discuss the advantages of integrating pre-trained networks into segmentation models and offer insights for future research in medical image analysis.

The remainder of this paper is organized as follows. Section 2 reviews related work on brain tumor segmentation and the application of deep learning models. Section 3 details the architecture of the proposed model and the methodological framework. Section 4 describes the experiments and results, including evaluation metrics, experimental setup, quantitative results, and ablation study. Section 5 presents the limitaion of this study. Sections 6 and 7 are discussion and future work, respectively. Finally, Section 8 concludes the paper and outlines potential directions for future work.

## Related work

This section examines the current research on brain tumor segmentation, emphasizing methodologies that utilize U-Net designs and incorporate pre-trained networks such as VGG-19. This study seeks to situate our proposed model within the wider research context, emphasizing both achievements and deficiencies. This study [20] strongly corresponds with our proposed methodology, which integrates a U-Net architecture with VGG-19 as a pre-trained model for brain tumor segmentation. Our approach aligns with the trend of automated and flexible segmentation models such as nnU-Net, prioritizing minimal manual involvement and enhanced performance. A notable example close to our work is a generative model that creates synthetic paired images and segmentation samples for supervised tasks [21]. These datasets can complement or replace real datasets while maintaining performance and improve out-of-distribution data performance [21]. This corresponds with our objective of improving brain tumor segmentation by utilizing pre-trained architectures such as U-Net with VGG-19, since both methodologies emphasize the progression of segmentation via creative strategies. Recent progress in brain tumor segmentation has incorporated deep learning architectures such as U-Net and U-Net++. It is noteworthy that it attains accuracy and Dice scores over 90% in the identification and segmentation of tumors from MRI data [22]. This study is relevant as it illustrates the efficacy of U-Net topologies in medical picture analysis [22]. Our research expands upon this foundation by including a pre-trained VGG-19 network into the U-Net architecture to augment feature extraction and enhance segmentation precision. A recent work introduced a completely automated approach for the detection and segmentation of brain tumors. This work utilizes single-spectral MRI data, with great accuracy and little computing complexity [23]. This study emphasizes the significance of effective segmentation utilizing readily available MRI data, which corresponds with our findings. Our methodology integrates a pre-trained U-Net with VGG-19, so augmenting segmentation efficacy on single-spectral MRI. It also diminishes dependence on multi-spectral data, and enhancing clinical applicability [23].

A new work [24] introduces SLCA-UNet, a sophisticated UNet model for brain tumor segmentation, which incorporates residual dense blocks and attention modules to improve feature extraction and precision on MRI data. Our research further develops UNet by utilizing a VGG19 backbone through transfer learning. Both approaches make use of UNet and ours through the extraction of pre-trained features. A new research [25] presents Tumor Bagging, a framework that combines many Multilayer Perceptron (MLP) models to enhance brain tumor segmentation. It is synthesized via majority voting and optimized using three metaheuristic algorithms: Spider Monkey Optimization, Artificial Electric Field Optimization, and Gray Wolf Optimization. It achieves a DSC of over 92% for thorough tumor detection using three MRI modalities. By using pre-trained CNNs, our VGG19-based U-Net with transfer learning, on the other hand, generates better metrics and increases segmentation accuracy.

VcaNet [26] presents an innovative architecture for 3D brain tumor segmentation from MRI data, using a Vision Transformer with a fusion channel and a spatial attention module (CBAM) to improve the integration of local and global features. While VcaNet utilizes transformers to capture long-range dependencies, our research employs a VGG19-based U-Net, applying transfer learning to attain high segmentation accuracy. A new study [27] presents a brain tumor segmentation technique that integrates a U-Net with a 3D CNN, employing GLCM for feature extraction and dual-network integration, attaining superior performance (mean accuracy 99.40%, precision 99.41%, F-Score 99.40%) on validation datasets. Conversely, our study utilizes a VGG19-based U-Net with transfer learning, producing robust outcomes (AUC 0.9957, Dice 0.9679). Both methodologies improve segmentation precision, ours through the utilization of pre-trained structures.

While existing methods like nnU-Net have made substantial contributions in automating and adapting segmentation models, they often require extensive computational resources and may not fully exploit the feature extraction capabilities of pre-trained networks like VGG19. Additionally, approaches utilizing generative models for data augmentation primarily focus on enhancing datasets but may not address the optimization of segmentation architectures for 2D brain tumor images specifically. Other techniques that use single-spectral MRI images tend to rely on handcrafted features and conventional classifiers, which might not capture the complex patterns necessary for precise tumor segmentation. Our research addresses these limitations by developing a 2D brain tumor segmentation model that employs VGG19 as an encoder within a U-Net architecture. By exploiting the feature representations from VGG19, our model enhances segmentation accuracy without the need for multi-spectral data or extensive computational resources. Our model demonstrates excellent performance in evaluation results. This approach not only fills the gaps left by previous studies but also provides a more efficient and accessible solution for accurate brain tumor detection. Table 1 provides a comprehensive summary of the key related works reviewed in this study. It highlights the objectives and limitations of each paper, offering a clear understanding of the context and gaps addressed by previous research.

**Table 1:** Summary of related works

Author(s)	Year	Objects	Limitation	Title
Isensee et al. [20]	2021	Brain tumor segmentation in MRI	High computational resources required	nnU-Net: a self-configuring method for deep learning-based medical image segmentation
Virginia Fernandez et al. [21]	2024	Multi-pathological 2D/3D brain MRI images	Mostly synthetic datasets; limited real-world data	Generating multi-pathological and multi-modal medical image data
Kashfia Sailunaz et al. [22]	2023	2D/3D brain MRI images	Need for dynamic user feedback to enhance model performance	Brain tumor detection and segmentation: Interactive learning framework
Gupta, A., et al. [23]	2020	Gabor Texture Features for 2D MRI Brain Tumor Detection	Utilized Gabor texture features in conjunction with machine learning	May not perform as well as deep learning methods
Tejashwini et al. [24]	2025	SLCA-UNet, an enhanced version of the UNet model to improve brain tumor segmentation	significant computational resources for training and deployment	A novel SLCA-UNet architecture for automatic MRI brain tumor segmentation
Shiv Naresh et al. [25]	2021	Brain tumor segmentation enhancement by a novel ensemble framework	Specific metaheuristic optimization algorithms chosen	Tumor bagging: a novel framework for brain tumor segmentation using metaheuristic optimization algorithms
Dichao Pan et al. [26]	2025	Propose VcaNet, a novel architecture that integrates a Vision Transformer (ViT) with a fusion channel and spatial attention module (CBAM)	High computational demand, a common challenge in Transformer-based models	VcaNet: Vision Transformer with fusion channel and spatial attention module for 3D brain tumor segmentation
Surendran R. et al. [27]	2023	An automated brain tumor segmentation method using a 3D CNN, GLCM, and VPT development	Hinder in real-time application and generalizability in resource-limited settings	Automated Segmentation of Brain Tumor MRI Images Using Deep Learning

## Methodology

This section describes the comprehensive methodologies used to develop and evaluate our deep learning model for brain tumor segmentation using MRI scans. It provides a detailed overview of the data acquisition process, preprocessing techniques, architecture of the proposed U-Net model with VGG19 as a pre-trained encoder, training procedures, evaluation metrics, and experimental setup. The goal is to establish a clear and reproducible framework that advances the field of medical image segmentation.

### Data acquisition

**Fig. 1:**
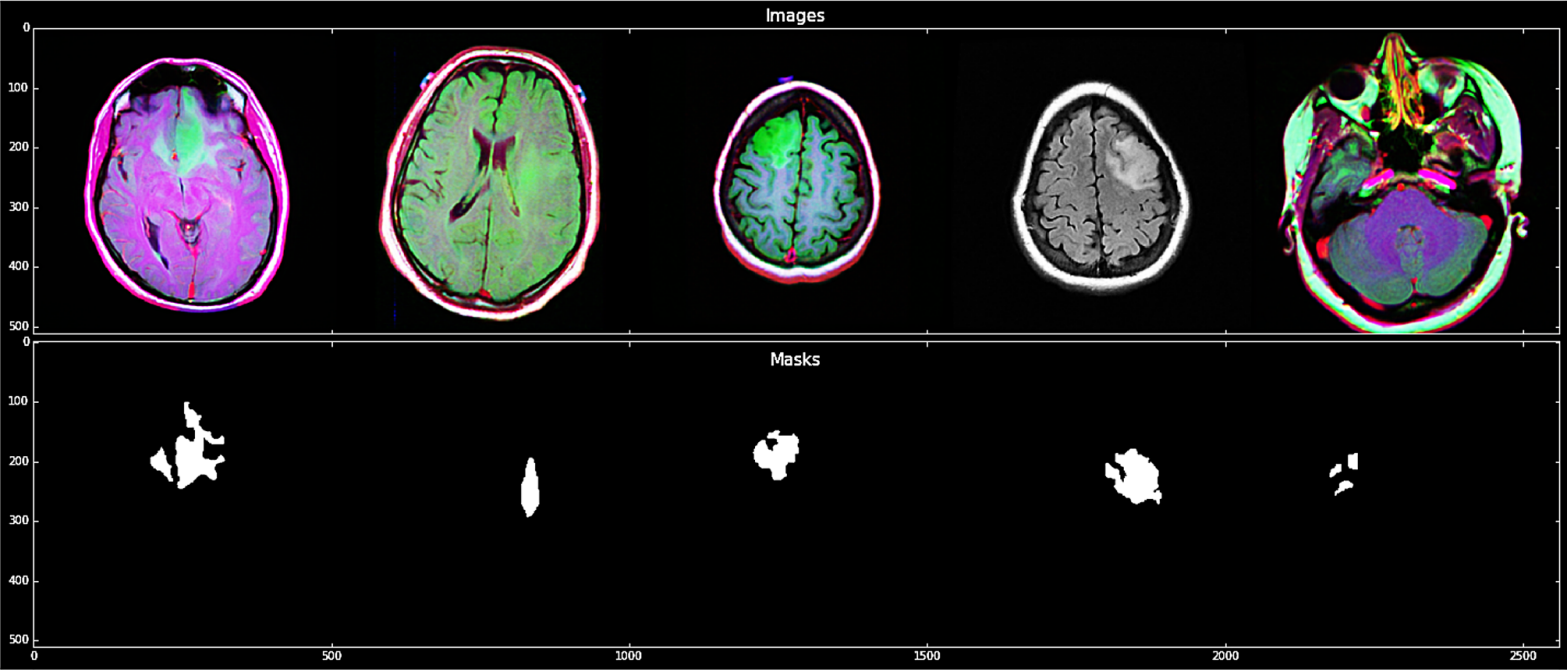
Representative examples of brain MRI images (top) and their corresponding ground truth masks (bottom) used for tumor segmentation

For this study, we used medical imaging data obtained from The Cancer Imaging Archive (TCIA) [16, 17], focusing on patients included in The Cancer Genome Atlas (TCGA) Lower-Grade Glioma (LGG) collection. The TCIA is a publicly accessible repository that offers an array of cancer imaging datasets, facilitating research and development in medical imaging and oncology. The dataset comprises MRI scans from 120 patients diagnosed with lower-grade gliomas, classified as Grade II and III. These tumors are known for their infiltrative nature and variable progression, making accurate segmentation essential for treatment planning. All patients in the dataset have at least FLAIR MRI sequences available. FLAIR imaging is instrumental as it suppresses cerebrospinal fluid signals, enhancing the visibility of lesions adjacent to the ventricles and within the cerebral cortex. While the dataset provides a strong foundation, the relatively small sample size of 120 patients may limit the model’s generalizability to broader populations or rare tumor subtypes. The TCIA TCGA-LGG dataset provides a valuable resource for developing brain tumor segmentation models. Its combination of high-resolution FLAIR MRI scans and associated genomic data offers a comprehensive platform for integrating imaging and molecular diagnostics, contributing to personalized patient care. The study utilizes the available Multimodal Brain Tumor Segmentation [16, 17]. The Fig. 1 illustrates different brain slices, highlighting the regions of interest where tumor boundaries have been segmented using the U-Net model with VGG19 as a pre-trained backbone.

**Fig. 2:**
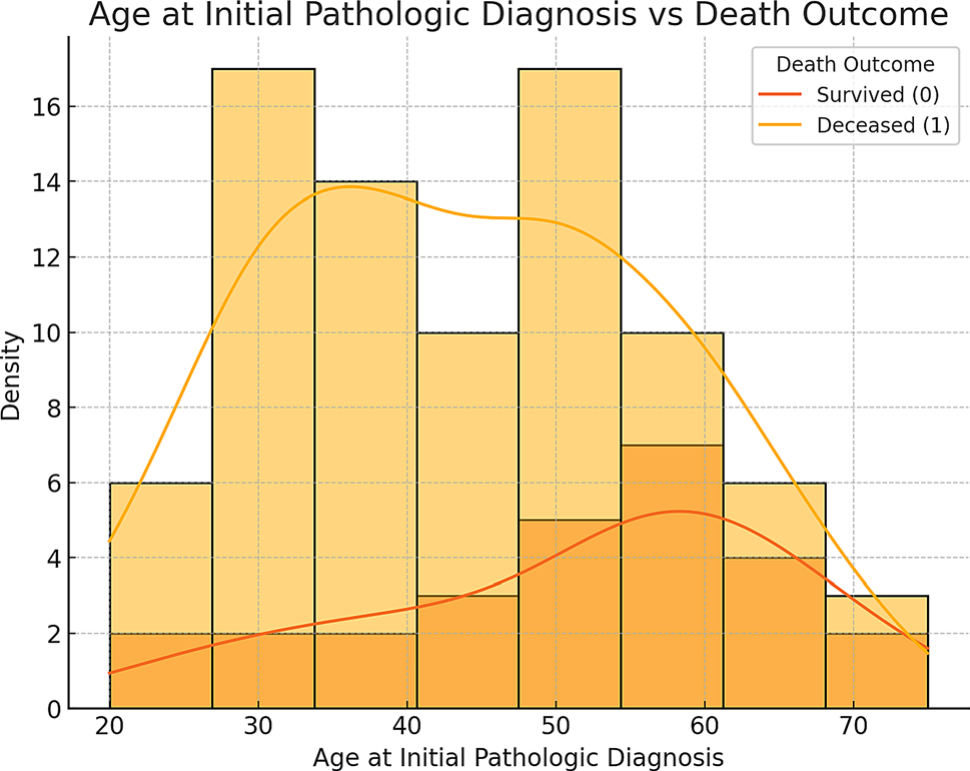
Age distribution at initial pathological diagnosis in relation to death outcome

Figure 2 demonstrates that survival outcomes for patients with lower-grade gliomas are linked to their age at diagnosis. Survival probability decreases with advancing age, with younger patients showing higher survival rates. Most patients were diagnosed between ages 30 and 50, but a higher density of deaths is observed in the 30 to 40 age range. These findings emphasize the importance of early detection and suggest that age-specific treatment strategies could improve survival across different age groups.

### Preprocessing steps

In developing the U-Net model for brain tumor segmentation, data preprocessing is essential to ensure the model receives properly formatted and standardized input. The preprocessing pipeline includes data splitting, image resizing, standardization, normalization, and efficient batch generation using a custom data generator. Below are the main components of the preprocessing code and their roles in preparing the dataset for model training.

To assess model performance and prevent overfitting, the dataset was divided into training, validation, and test sets. This split was performed using the train-test-split function from scikit-learn, following these steps:

The dataset was initially split into a training set and a combined validation-test set with a 15% test size. The combined validation-test set was then further divided equally to obtain separate validation and test sets.

Additionally, a custom data generator handled the preprocessing tasks, including resizing, standardization, batch generation with shuffling, and mask binarization.

### Model architecture

The U-Net architecture, a symmetric encoder-decoder network, is specifically designed for biomedical image segmentation. The encoder captures context through successive convolutional and max-pooling layers, reducing spatial dimensions while increasing feature depth. In contrast, the decoder performs upsampling and convolution operations to restore spatial dimensions and refine segmentation details. An overview of the U-Net architecture is discussed in detail later in this section.

#### Skip connections

**Fig. 3:**
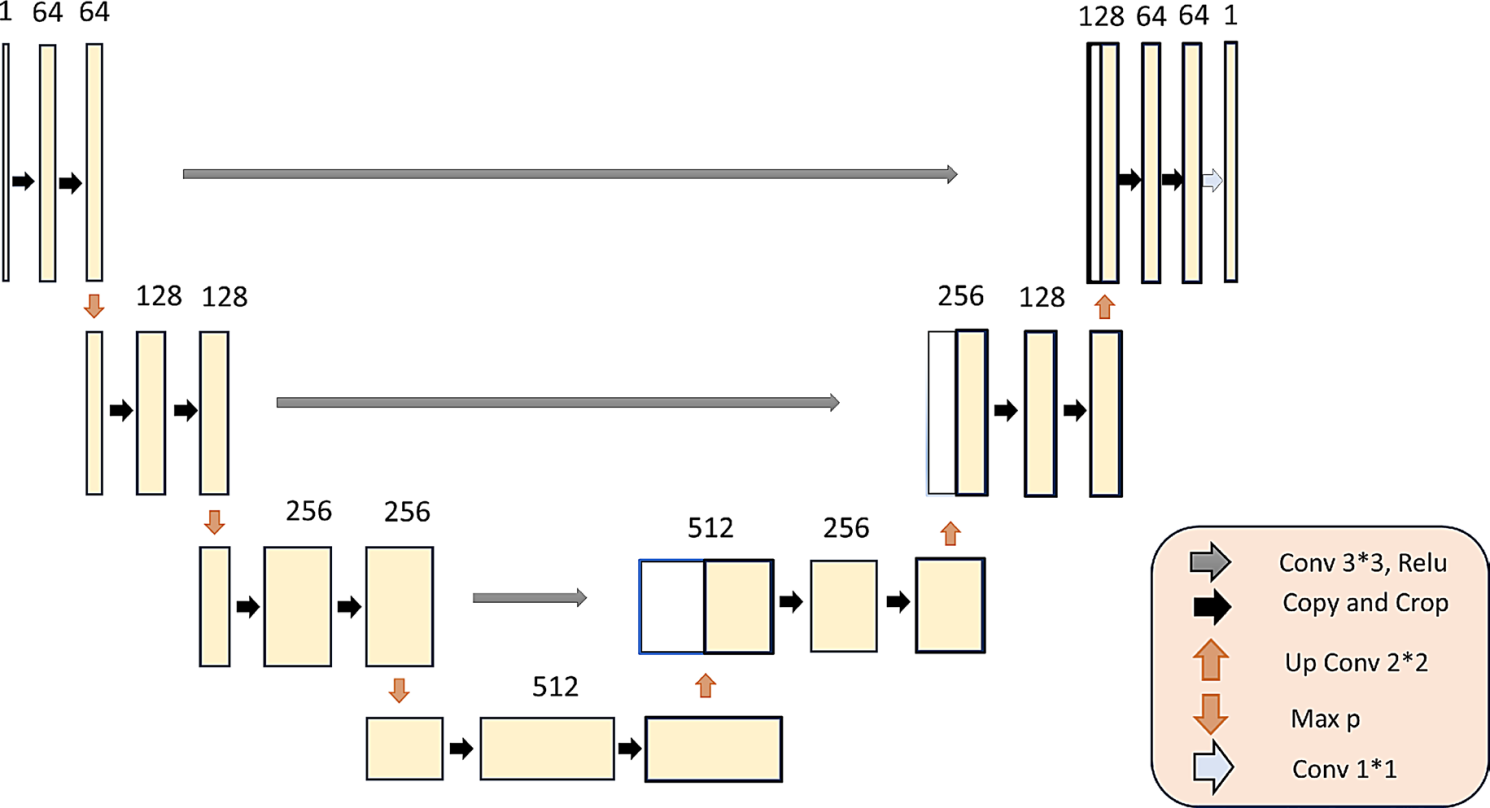
U-net architecture diagram

Skip connections link corresponding layers in the encoder and decoder paths, directly transferring high-resolution features from the encoder to the decoder. This mechanism preserves spatial information lost during downsampling, enhancing localization accuracy. Figure 3 illustrates the specific U-Net architecture used in our model.

#### Proposed method

This study presents a deep learning approach for brain tumor segmentation in MRI images by integrating a U-Net architecture with a pre-trained VGG19 model as the encoder. This method uses the strengths of both architectures: VGG19’s strong feature extraction and U-Net’s effective upsampling and localization capabilities. By combining these two models, the approach aims to accurately identify and segment tumor regions within brain MRI scans.

The encoder uses the convolutional layers of the VGG19 model, which has been pre-trained on the ImageNet dataset. By importing VGG19 without its fully connected layers, the model benefits from learned features that capture essential visual patterns. This transfer learning approach enables the encoder to extract rich, hierarchical features from the input MRI images, enhancing the models capacity to recognize complex structures associated with brain tumors.

To employ VGG19’s powerful feature extraction, pre-trained on ImageNet, its convolutional layers were integrated as the encoder component of the U-Net.

The decoder reconstructs the segmentation mask from the high-level features extracted by the encoder. Using transposed convolutional layers (also known as deconvolution layers), it upscales the feature maps, progressively restoring the spatial dimensions reduced during encoding. To preserve vital spatial information and refine segmentation boundaries, skip connections concatenate feature maps from corresponding encoder layers directly to the decoder layers, allowing simultaneous access to low-level and high-level features.

Both encoder and decoder employ convolutional blocks, each consisting of convolutional layers followed by batch normalization and activation functions. Batch normalization standardizes the inputs to each layer, promoting faster convergence and improved generalization by reducing internal covariate shifts. The activation function used is the Rectified Linear Unit (ReLU), which introduces non-linearity, allowing the model to capture complex patterns within the data. The model accepts input images with dimensions suited to the MRI scan resolution. The final layer applies a convolution operation with a sigmoid activation function, producing a probability map indicating each pixels likelihood of belonging to a tumor region. This results in a binary segmentation mask after thresholding.

**Fig. 4:**
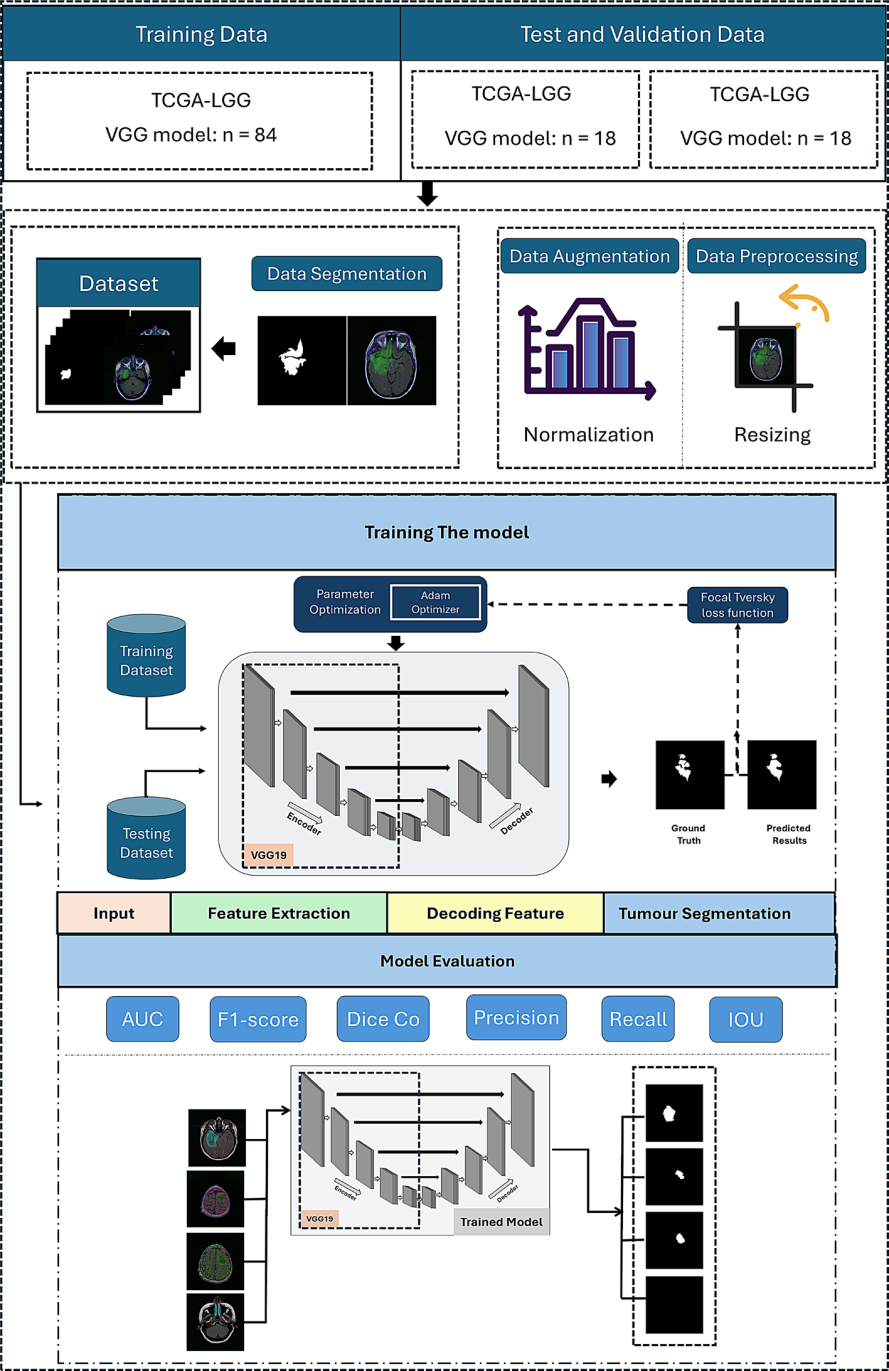
Framework of the proposed model leveraging VGG19 architecture

Figure 4 illustrates the framework of our proposed model.

Algorithm 1 presents the methodology for segmenting brain tumors from MRI images using a VGG19 U-Net architecture. The first step begins with the initialization of the model parameters specific to the VGG19 U-Net, which integrates the pre-trained VGG19 network as the encoder within a U-Net framework. Data augmentation and preprocessing steps such as resizing and normalization are applied to the MRI image dataset D to enhance the model’s generalization capabilities. The dataset is shuffled to ensure randomness during training. For each image in the shuffled dataset, feature extraction is performed. These features are then passed through decoder blocks with skip connections that merge encoder and decoder features. The loss between the predicted mask and the ground truth is calculated using the Focal Tversky loss function.

**Figure:**
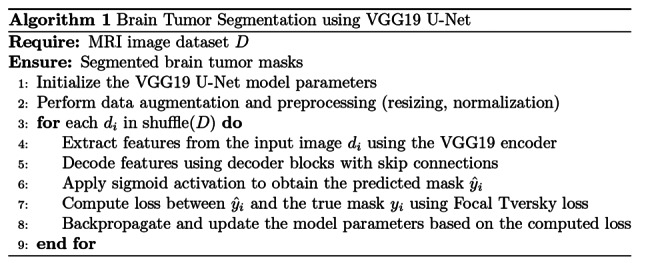

#### Loss function

To train our segmentation model, we used the Focal Tversky loss function, which is especially effective for managing class imbalances in segmentation tasks by focusing on hard examples. The Focal Tversky loss is derived from the Tversky index, a generalization of the Dice coefficient and Jaccard index, and is defined as follows:

where the Tversky index is given by:

1 $$\begin{aligned}& \mathcal{L}_{\text{Focal Tversky}} \cr&= \left(1 - \frac{\sum\limits_{i} p_i g_i}{\sum\limits_{i} p_i g_i + \alpha \sum\limits_{i} p_i (1 - g_i) + \beta \sum\limits_{i} (1 - p_i) g_i}\right)^\gamma\end{aligned}$$

where $\mathcal{L}_{\text{Focal Tversky}}$ is the loss, $p_i$ is the predicted probability for element $i$, $g_i$ is the ground truth label, $\alpha$ and $\beta$ are weighting factors for false positives and negatives, $\gamma > 0$ is the focusing parameter, and $\sum\limits_{i}$ sums over all elements. Minimizing this loss enhances the model’s ability to handle class imbalances by focusing on hard examples, thereby improving segmentation of the minority class. Our method, utilizing transfer learning and skip connections, captures key features for accurate brain tumor segmentation, showing promise for clinical diagnosis and treatment planning.

## Experiments and results

In this section, we present a comprehensive evaluation of our proposed brain MRI tumor segmentation model. The primary objective is to demonstrate the model’s effectiveness in accurately segmenting brain tumors from MRI scans. We compare its performance against seven other models that utilize different pre-trained CNNs as backbones, highlighting the strengths of our approach.

### Evaluation metrics

We evaluated our brain tumor segmentation model using standard metrics: AUC, Dice Coefficient, F1-Score, IoU, Precision, and Recall.

#### Area under the receiver operating characteristic curve (AUC)

The AUC measures the classifier’s ability to distinguish between classes across all thresholds.

2 $$ \text{AUC} = \int_{0}^{1} \text{TPR}(t), d\text{FPR}(t) $$

where $\text{TPR}(t)$ is the true positive rate at threshold $t$, $\text{FPR}(t)$ is the false positive rate at threshold $t$.

#### Dice coefficient

The Dice Coefficient quantifies overlap between predicted segmentation $X$ and ground truth $Y$.

3 $$ \text{Dice} = \frac{2 \times|X \cap Y|}{|X| +|Y|}$$

where $|X \cap Y|$ is the intersection, and $|X|$ and $|Y|$ are the set sizes.

#### F1-Score

The F1-Score is the harmonic mean of Precision and Recall.

4 $${\rm{F}}1 - {\rm{Score}} = 2 \times {{{\rm{Precision}} \times {\rm{Recall}}} \over {{\rm{Precision}} + {\rm{Recall}}}}$$

#### Intersection over union (IoU)

IoU, or Jaccard Index, measures overlap relative to the union.

5 $$\text{IoU} = \frac{|X \cap Y|}{|X \cup Y|}$$

where $|X \cap Y|$ is the number of elements common to both the predicted set $X$ and the ground truth set $Y$, $|X \cup Y|$ is the number of elements in the union of the predicted set $X$ and the ground truth set $Y$.

#### Precision

Precision is the proportion of correctly predicted positives.

6 $$\text{Precision} = \frac{TP}{TP + FP}$$

#### Recall

Recall measures the proportion of actual positives correctly identified.

7 $$\text{Recall} = \frac{TP}{TP + FN}$$

By utilizing these metrics, we aim to provide a coherent evaluation of our model’s performance, capturing not only its accuracy but also its reliability in correctly segmenting brain tumors across varying cases.

### Experimental setup

The hardware specifications for this study will be discussed in this section. The GPU of the system, used in this study, is NVIDIA RTX 3050 GPU, equipped with 4GB of VRAM. For central processing, the setup featured an AMD Ryzen 7 4800 H CPU, an eight-core processor. Additionally, the system was configured with 32GB of RAM.

Table 2 summarizes the key hyperparameters and protocols used during training across several models.

**Table 2:** Summary of hyperparameter settings and training protocols

Model	VGG-19	U-Net	FPN	Combined Model Class	Combined Model Seg
Learning Rate	0.05	0.001	Default	Default	0.05
Batch Size	32	40	26	16	16
Number of Epochs	60	80	30	50	60
Optimizer	Adam	Adamax	Adam	Adam	Adam
Loss Function	Focal Tversky Loss	Dice Loss	BCE Dice Loss (bce dice loss)	Categorical Crossentropy	Focal Tversky Loss

#### Dataset splitting

Our dataset was divided into training (70%), validation (15%), and test (15%) sets using stratified sampling. This approach maintained a consistent proportion of tumor-positive and tumor-negative cases across all subsets. This strategy was applied on all of the models that are trained in this research. All MRI images and segmentation masks were resized to a uniform 256 × 256 pixels.

### Quantitative results

Table 3 presents the comparative metrics for various models. This table summarizes the calculated evaluation metrics corresponding to each model.

**Table 3:** Comprehensive evaluation metric results

Model	AUC	F1-Score	Dice Co	Precision	Recall	IOU
VGG-19	0.9957	0.9679	0.9679	0.9541	0.9821	0.9378
Two-Stage model	0.954	0.9428	0.9129	0.8774	0.9547	0.8595
U-Net	0.9348	0.8778	0.8779	0.9043	0.8572	0.7853
FPN-efficientnet	0.9103	0.8633	0.9901	0.8633	0.9233	0.8905
FPN	0.9303	0.8633	0.8672	0.8622	0.8633	0.7605
InceptionV3	0.8523	0.8308	0.8284	0.8612	0.8442	0.7191
ResNet	0.9213	0.9215	0.9688	0.8704	0.9519	0.9123
ResNeXt	0.8315	0.8163	0.8236	0.8178	0.8031	0.6957

**Fig. 5:**
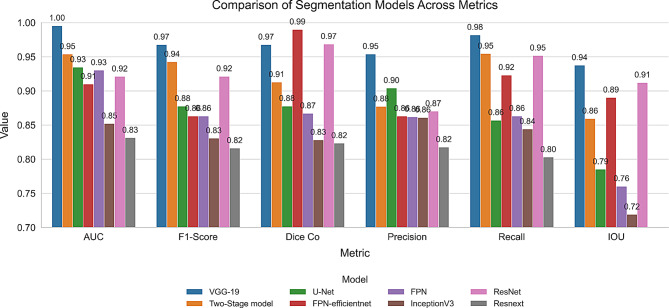
Comparison of segmentation models across key performance metrics

Figure 5 presents a bar chart summarizing the different metrics across various models for better comprehension.

**Fig. 6:**
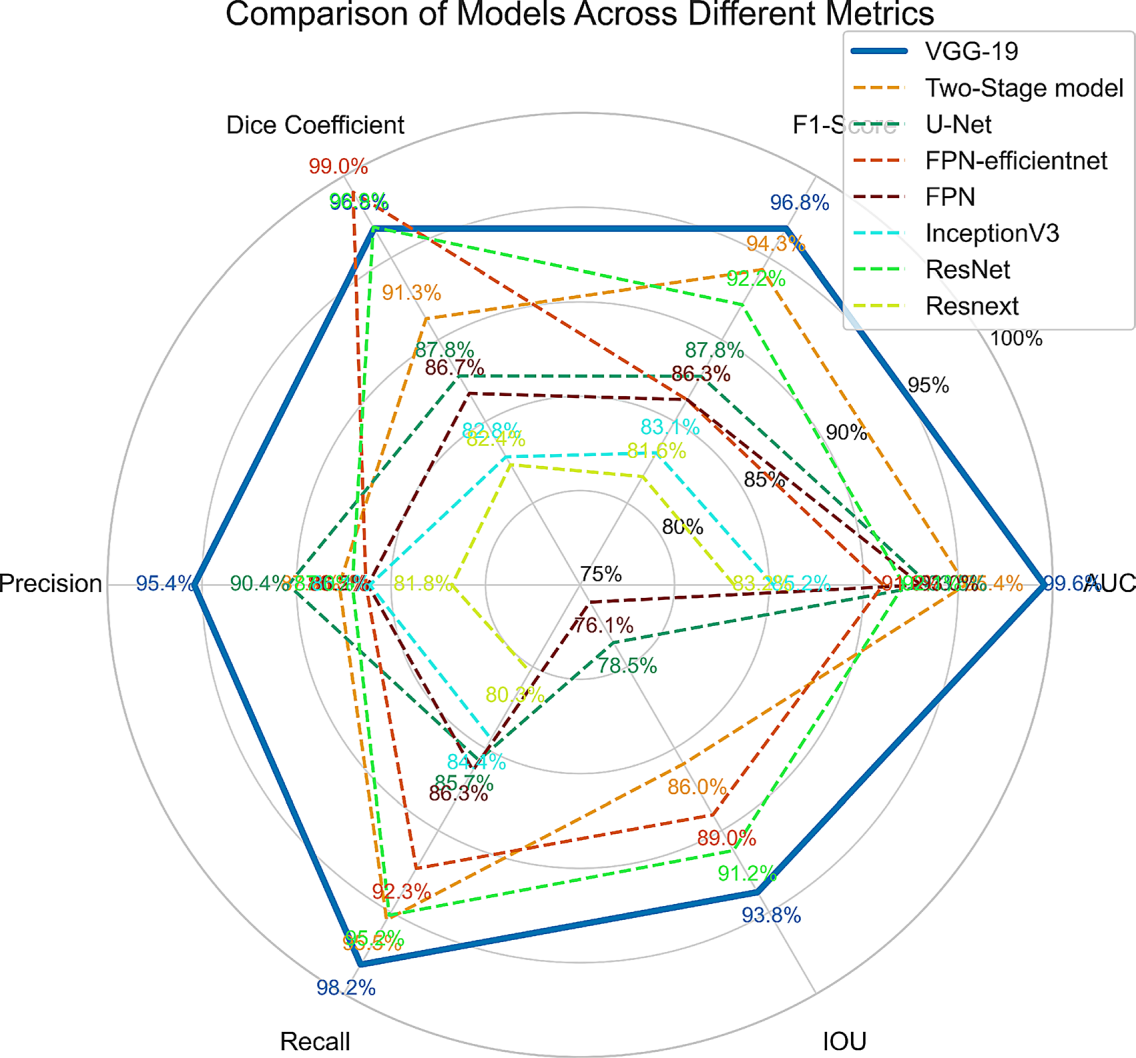
Comparative radar chart of segmentation models

**Fig. 7:**
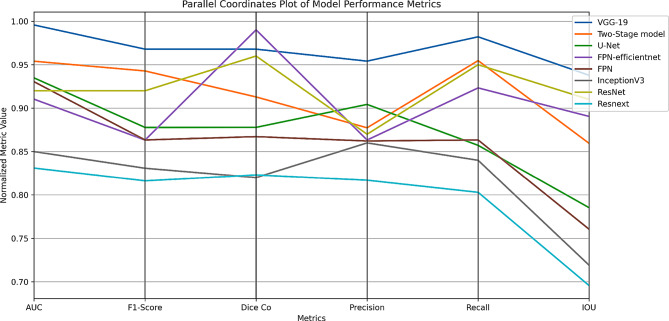
Comparative parallel coordinates plot

Figure 6 presents a radar chart which compares our proposed model with other models across six critical evaluation metrics. This radar chart provides a visual summary, highlighting the VGG-19 models robustness and overall advantage. Additionally, we provided a parallel coordinates plot in Fig. 7 to facilitate comparison.

**Fig. 8:**
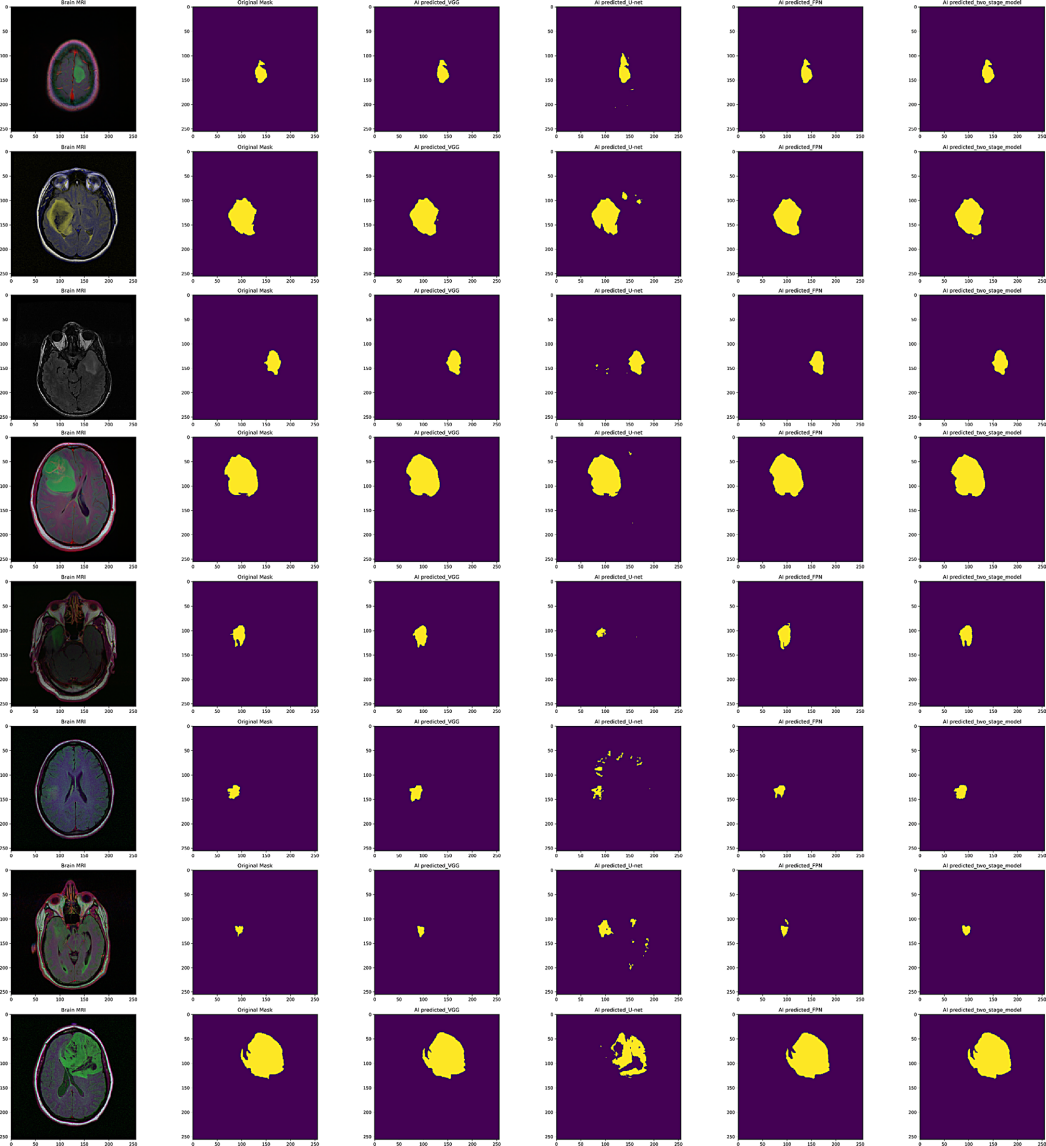
Brain MRI, original mask, AI predicted VGG-19, AI predicted U-net, AI predicted FPN-efficientnet, AI predicted two-stage model

Figure 8 illustrates the the brain MRI tumor alongside the predicted tumor regions, which are segmented by different models. This figure compares a brain MRI scan (as illustrated in the first column) with its ground truth mask (as illustrated in the second column) and AI-generated segmentation masks from VGG-19 (as illustrated in the third column), U-Net (as illustrated in the fourth column), FPN-EfficientNet (as illustrated in the fifth column), and a two-stage model (as illustrated in the sixth column), showcasing their performance in delineating brain structures.

**Fig. 9:**
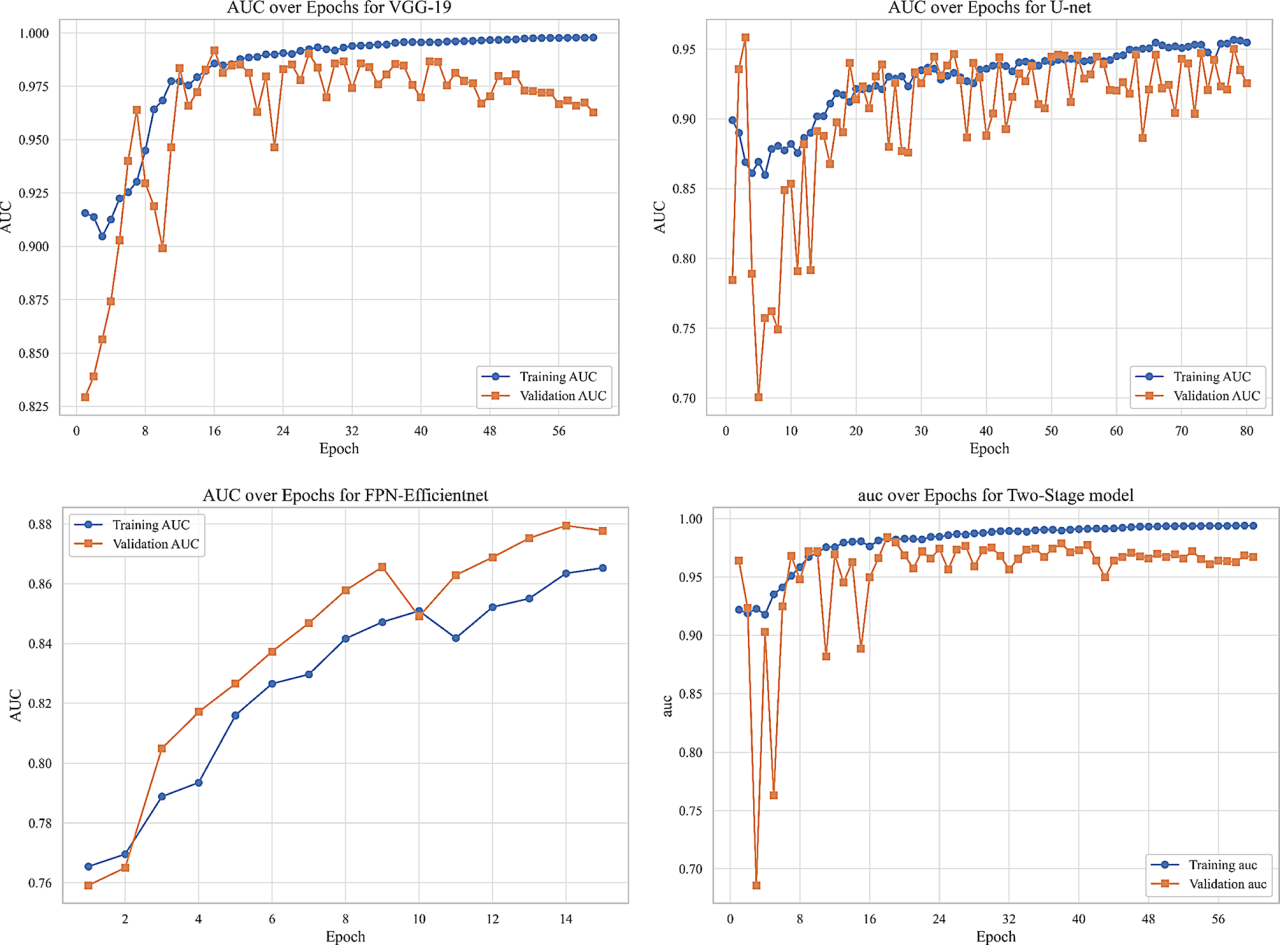
AUC curve over epochs illustration across various models

**Fig. 10:**
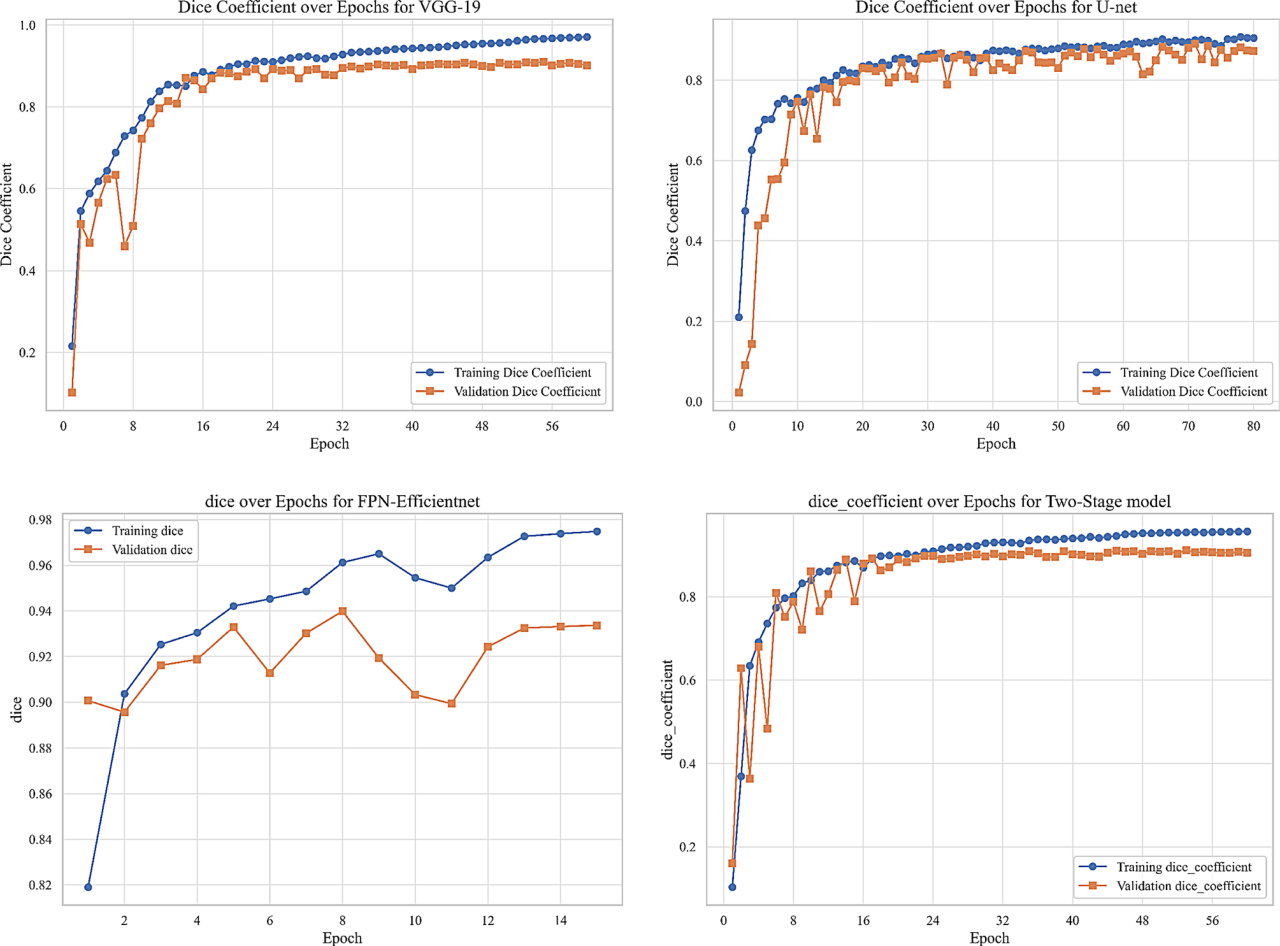
Dice coefficient curve over epochs illustration across various models

**Fig. 11:**
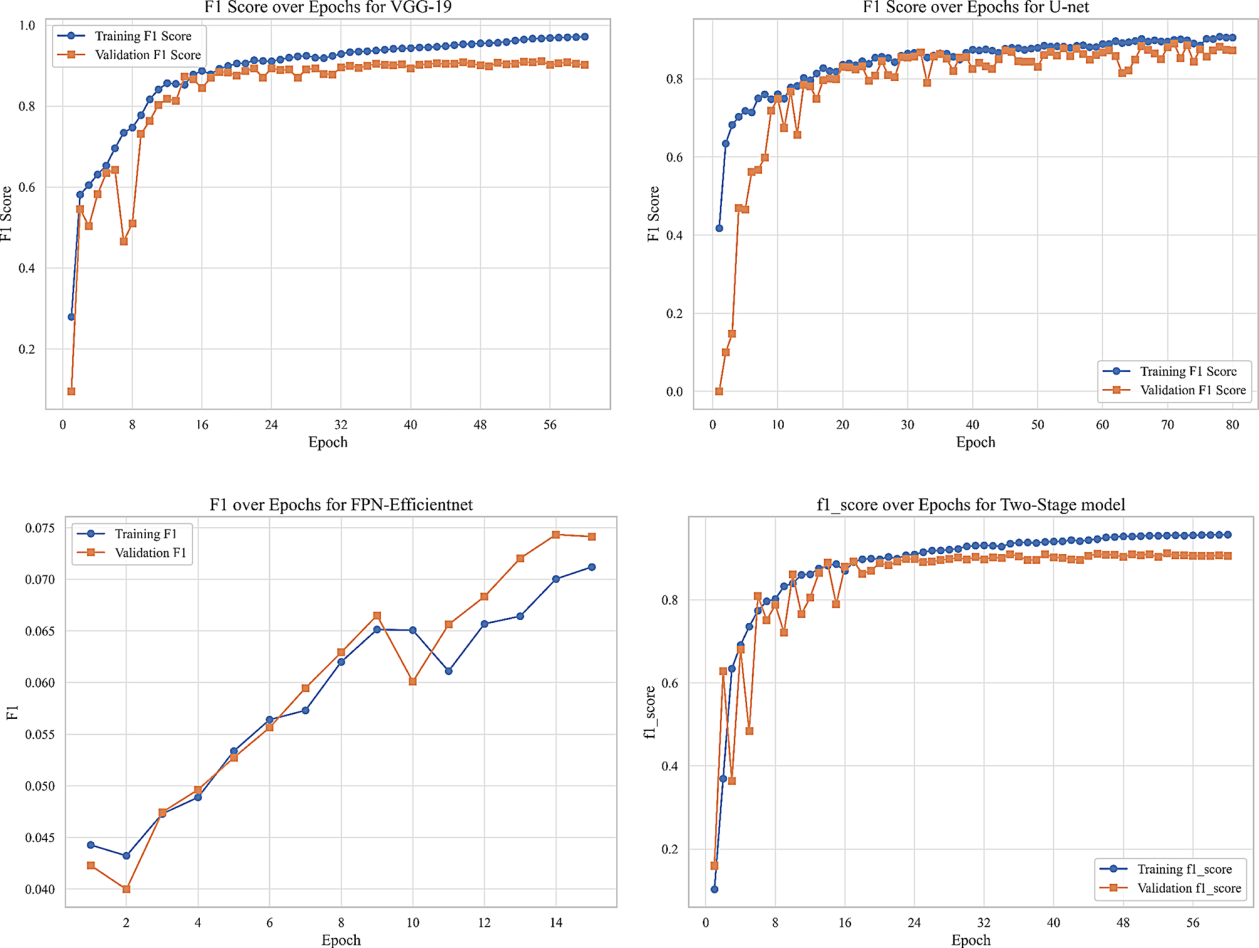
F1-score curve over epochs illustration across various models

**Fig. 12:**
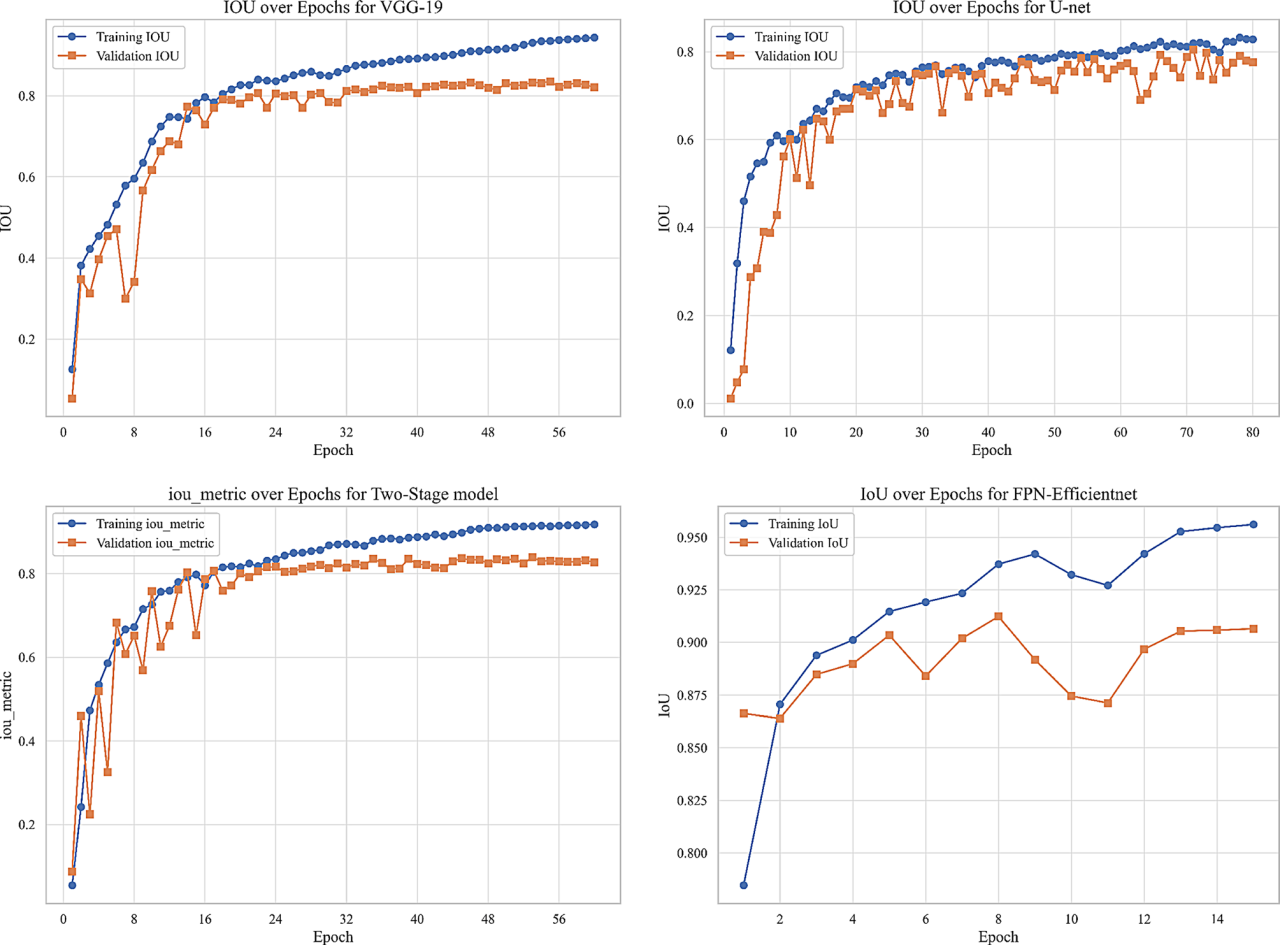
Intersection over union (IoU) curve over epochs illustration across various models

**Fig. 13:**
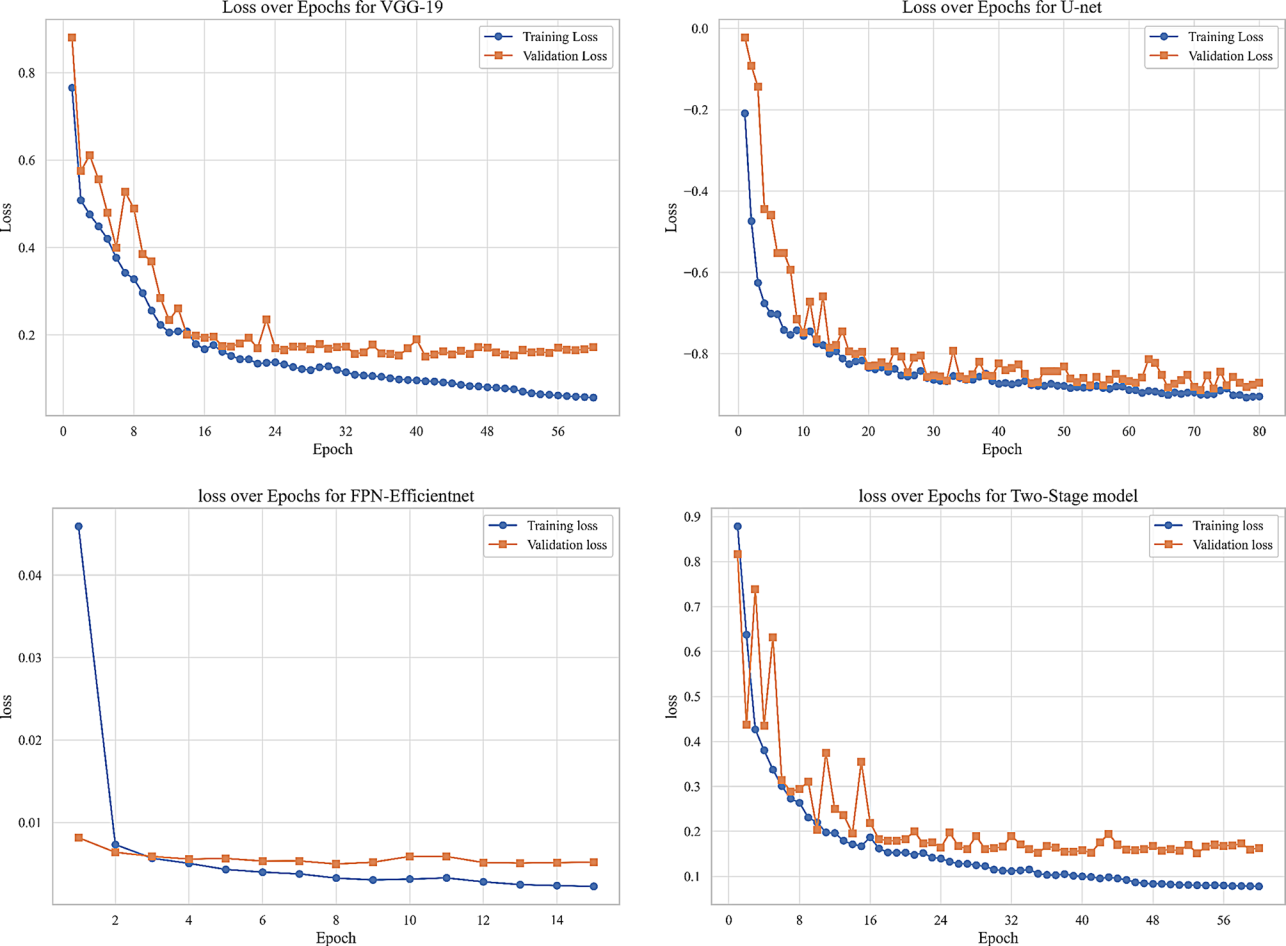
Loss curve over epochs illustration across various models

**Fig. 14:**
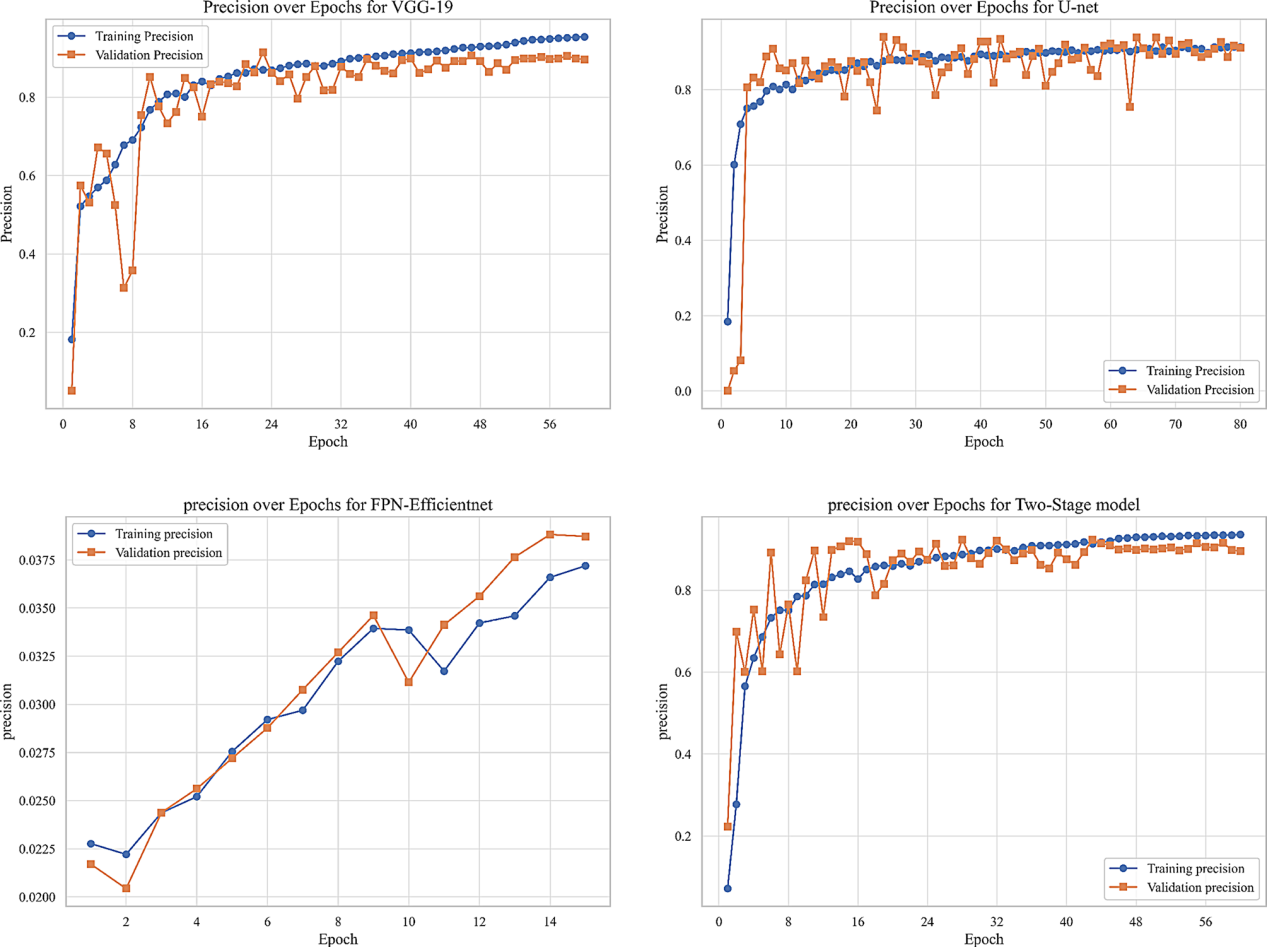
Precision curve over epochs illustration across various models

We evaluated our model using six metrics: Dice coefficient, IoU, precision, recall, F1-score, and AUC. Figures 9 to 14 displays the progression of training and validation metrics over epochs. They showcase the various performance metric curves central to this study. Figure 9 illustrates the AUC progression over training epochs. It enables a comparative assessment of classification performance across different models. Figure 10 highlights the evolution of the Dice coefficient. It reflects how effectively each model captures overlap in segmentation tasks. In Fig. 11, the F1-score curve demonstrates the balance between precision and recall over epochs, while Fig. 12 focuses on the IoU metric to measure segmentation accuracy. Figure 13 showcases the loss curves. Finally, Fig. 14 presents the precision curves.

As illustrated, all metrics show an initial rapid improvement during the initial epochs. The fluctuations observed in the validation precision may be due to the limited size of the validation dataset or class imbalance. Overall, the training and validation curves suggest that our proposed model achieves strong segmentation performance, which could improve diagnostic accuracy in clinical settings.

### Ablation study

To thoroughly investigate the impact of different components and hyperparameters in our proposed model, we performed an ablation research by methodically altering the baseline architecture (Full Model with VGG-19 backbone) and evaluating the resultant performance. We examine the impact of excluding skip connections, simplifying the backbone to VGG-16, maintaining partial skip connections, modifying the loss function to Dice or IOU loss, and adjusting critical hyperparameters (dropout and learning rate). The inclusion of skip connections in the baseline model led to a significant improvement in all metrics: AUC increased by approximately 2.60%, F1-score by 13.63%, Precision by 20.77%, Recall by 6.30%, Dice coefficient by 13.63%, and IOU by 26.42% compared to the model without skip connections. Substituting the VGG-19 backbone with the more simplistic VGG-16 architecture resulted in a significant decline in performance: AUC decreased to 0.9 (−9.61%), F1-score to 0.6764 (−30.15%), Precision to 0.6764 (−29.10%), Recall to 0.6994 (−28.79%), Dice coefficient to 0.6764 (−30.15%), and IOU to 0.511 (−45.52%). This significant decrease indicates the diminished representational capability of VGG-16, which possesses fewer convolutional layers (13 compared to 16 in VGG-19). Moreover, maintaining solely partial skip connections resulted in an AUC of 0.8782 (−11.80%), an F1-score of 0.6489 (−32.98%), a Precision of 0.5772 (−39.49%), a Recall of 0.741 (−24.52%), a Dice coefficient of 0.6489 (−32.98%), and an IOU of 0.4803 (−48.80%). This variation demonstrates a greater reduction in AUC, F1-score, and IOU compared to the complete exclusion of skip connections, although exhibits an increased Recall (+0.741 vs. 0.9239). This indicates that partial skip connections maintain some capacity to identify true positives, albeit with a substantial reduction in Precision, signifying an escalation in false positives. The decline of the IOU below that of the “No Skip Connections” variation underscores that incomplete skip connections more significantly disturb the equilibrium of feature propagation than their total absence, perhaps due to erratic information flow across layers.

Switching the loss function from the baseline’s Focal Tversky loss to Dice loss resulted in an decrease in metrics as well. Although Dice loss is intended to enhance overlap-based metrics such as the Dice coefficient, its sole application in this context resulted in a substantial decline in metrics. Similarly, employing IOU loss instead of Focal Tversky loss resulted in another decrease. The ablation investigation demonstrates that the baseline model’s exceptional performance (AUC: 0.9957, F1-score: 0.9679, IOU: 0.9378) is significantly dependent on the integration of its elements: VGG-19 backbone, comprehensive skip connections, and a Focal Tversky loss function. For the parameter varied model, we rose the dropout rate from 10 to 20 and also chose a lower learning rate of 0.0001. These also resulted in a lower performance metrics. Consequently, the ablation investigation confirms the architectural and optimization decisions of the baseline model, indicating that alterations in structure, loss, or hyperparameters consistently impair performance in both segmentation and classification measures. Table 4 is illustrating the comparative evaluation metrics for various models, enabling the comparison to the baseline model.

**Table 4:** Comprehensive ablation evaluation metric results

Model	AUC	F1-Score	Dice Co	Precision	Recall	IOU
Full Model (Baseline)	0.9957	0.9679	0.9541	0.9821	0.9679	0.9378
No Skip Connections	0.9705	0.8518	0.79	0.9239	0.8518	0.7418
Simpler Backbone (VGG-16)	0.9	0.6764	0.6764	0.6994	0.6764	0.511
Partial Skip Connections	0.8782	0.6489	0.5772	0.741	0.6489	0.4803
Model with Dice loss	0.828	0.6751	0.7292	0.6284	0.6751	0.5095
Model with IOU loss	0.7852	0.6722	0.7701	0.5964	0.6722	0.5063
Model with parameters varied	0.9287	0.5111	0.5266	0.7279	0.6111	0.4399

**Fig. 15:**
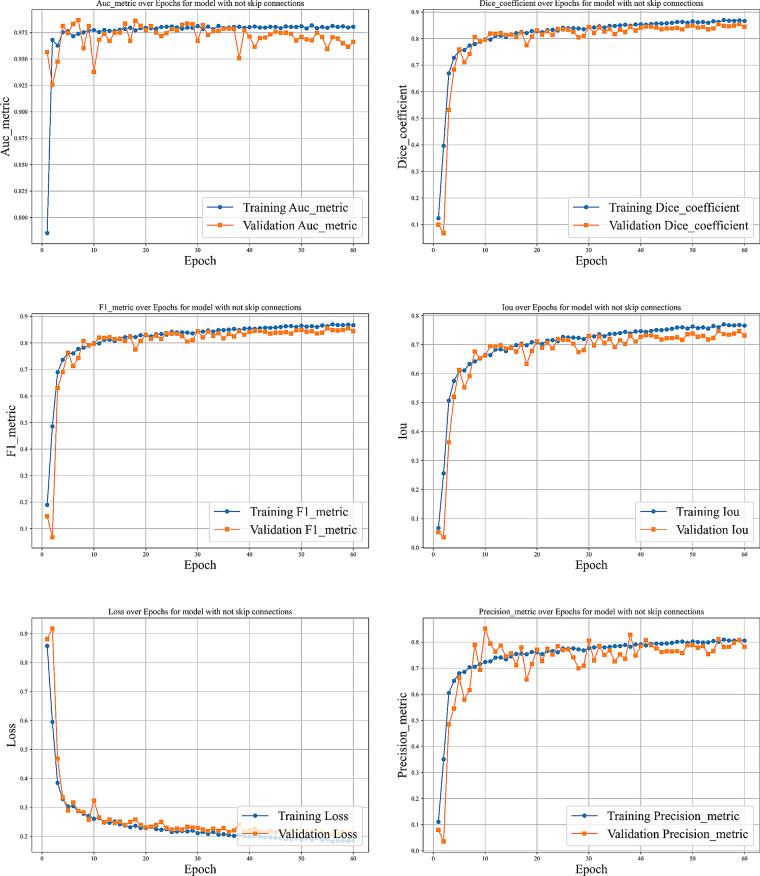
Different curves of training the model with no skip connections

**Fig. 16:**
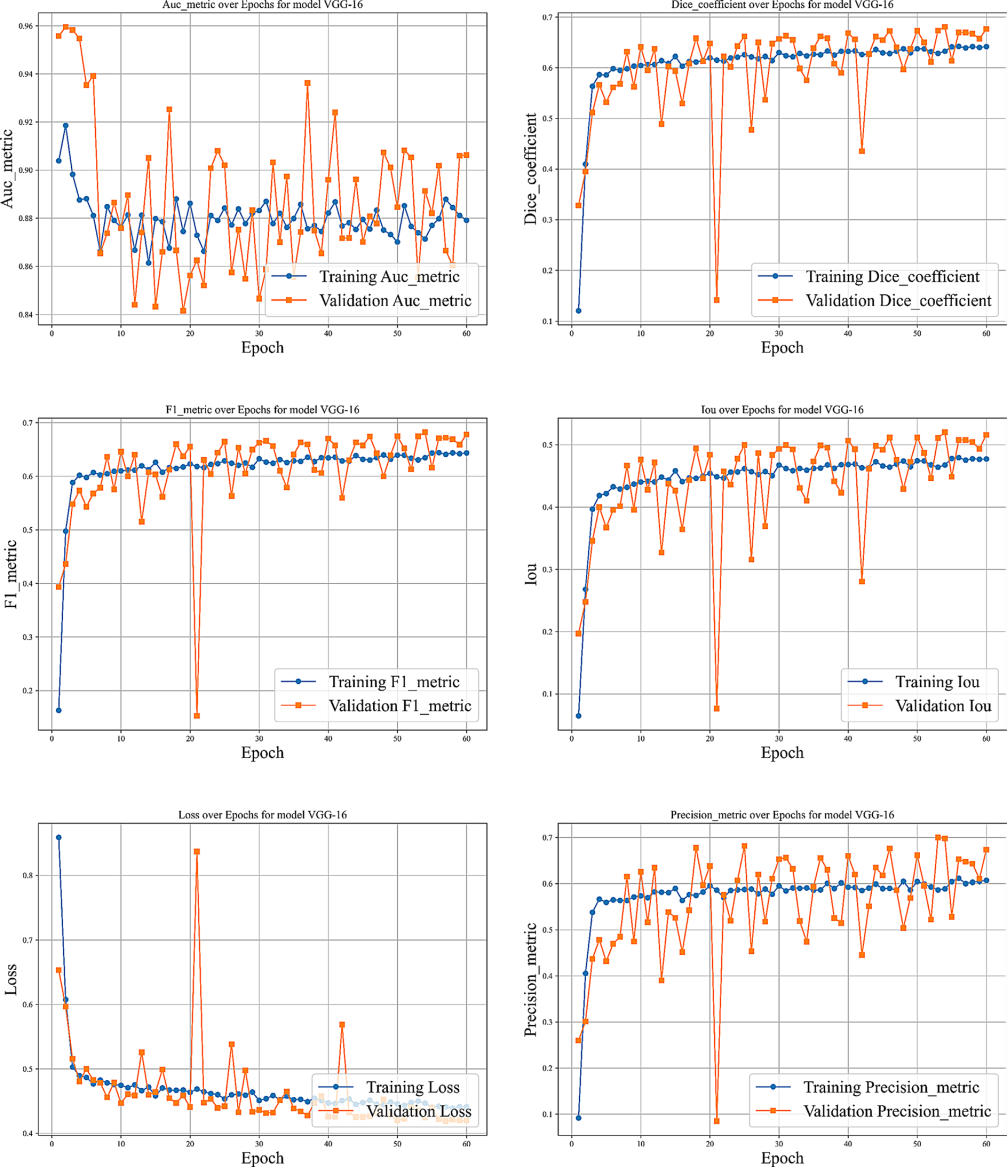
Different curves of training the VGG-16 model

**Fig. 17:**
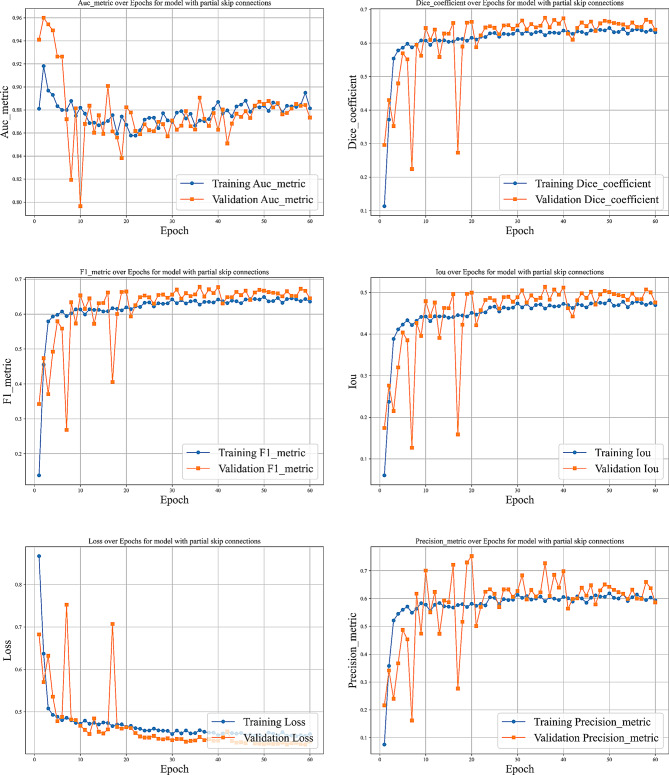
Different curves of training the model with partial skip connections

Figures 15–17 depict the performance curves across epochs for the model with skip connections omitted, the model using VGG-16 as a backbone, and the model with partial skip connections, respectively.

## Limitation

Although the proposed U-Net model with transfer learning shows promising results in brain tumor segmentation, this method still has some limitations. The model was trained and tested on a dataset, which may not fully reflect the variety of tumor types, sizes, and patient characteristics seen in real clinical settings. The experiments also used two-dimensional MRI slices instead of three-dimensional data, which may reduce the spatial accuracy of the segmentation. In addition, the model was tested only on single-modality MRI images, while using multimodal scans could improve the results. These factors may limit how well the model works in real-world situations. Future work should focus on addressing these issues to make the model more useful in practice. Additionally, we intend to utilize the BraTs dataset, namely BraTs 2020, 2021, etc., to train a 3D model.

## Discussion

Our research’s main goal was to improve the accuracy of brain tumor segmentation in MRI images by utilizing a custom Focal Tversky loss function and using VGG-19 as the encoder of the U-net architecture. According to our experimental findings, the proposed model performs better in terms of performance metrics than both conventional U-Net models and other models that use other pre-trained backbones. Incorporation of the pre-trained VGG19 encoder into the U-Net architecture has assited the feature extraction capabilities of the model. In order to address the class imbalance present in medical imaging segmentation tasks, the custom Focal Tversky loss function was considred. By adjusting the balance between false positives and false negatives through the Tversky index parameters ($\alpha$ = 0.7, $beta$ = 0.3) and emphasizing hard-to-classify pixels with the focal parameter ($gamma$ = 0.75), the loss function effectively penalized misclassifications that could lead to missed tumor regions. This leads to higher recall values, indicating the model’s improved sensitivity to tumor detection. As illustrated in Fig. 5, our model attained a Dice coefficient of 0.96, exceeding the performance of the other models. The IoU scores exhibited a comparable pattern. The AUC score of 0.99 indicates the model’s strong capacity to differentiate between tumor and non-tumor regions across various threshold settings. The models utilizing deeper or more advanced encoders, including VGG-19, ResNet, and FPN-efficientnet, generally demonstrate superior performance across the majority of metrics. In particular, the VGG-19-based approach shows a near-perfect AUC score (0.99) and consistently strong results in F1-Score (0.96), Dice-coefficient (0.96), and Precision (0.95). ResNet-based and FPN-efficientnet-based architectures likewise perform competitively, especially in metrics closely tied to segmentation accuracy (e.g., Dice, IoU). The steeper lines between adjacent axes in Fig. 7 of the parallel coordinates signify more pronounced shifts in performance for a specific model. Our proposed model consistently demonstrates high performance across most metrics. In contrast, other models demonstrate substantial reductions in particular metrics, indicating potential trade-offs—favoring recall at the expense of precision, or vice versa. In Fig. 6, our radar chart, the outer boundaries of each axis indicate that the VGG-19 model (solid blue) is positioned near or at the outer edge on several metrics (AUC, Precision, Recall, IoU), implying a strong overall performance. Meanwhile, the Two-Stage model (orange dashed line) stands out particularly at F1-Score and Dice Coefficient, implying it is particularly adept at capturing the correct proportion of positive pixels (F1) and generating high overlap with the ground truth segmentation masks (Dice). The model’s robust performance on the TCGA lower-grade glioma dataset indicates its capacity to efficiently expand to larger datasets or analogous tasks without necessitating substantial retraining.

## Conclusion and future work

Given the obtained result from experimental results and tests, it seemed that the proposed U-Net model, using a VGG19 backbone and a tailored Focal Tversky loss function, may provide improvements in brain tumor segmentation compared to traditional models and other U-Net variations employing other pre-trained encoders. Our presented method can enhance automated brain MRI image segmentation by potentially achieving high segmentation precision. Our method can advance automated brain MRI image segmentation by potentially attaining high segmentation accuracy. Our model’s quantitative results include an AUC of 0.9957, F1-Score of 0.9679, Dice Coefficient of 0.9679, Precision of 0.9541, Recall of 0.9821, and IoU of 0.9378. These may highlight the potential strength and therapeutic significance of the proposed model. To extend the current framework in the future, considering multimodal MRI scans, 3-dimensional volumetric segmentation, and larger patient populations can become closer to real-world application needs. Ultimately, ongoing advancements in deep learning for medical imaging could produce diagnostic tools that are more precise, efficient, and accessible, thereby enhancing patient outcomes. Our future research directions will include a number of key tactics to overcome the limitations mentioned before and improve the model’s capabilities, such as dealing with multimodal MRI scans. We consider solving the model’s generalizability and robustness by growing the dataset by gathering and integrating a larger dataset, such as different tumor types and sizes. Moreover, the integration of multimodal imaging has the potential to enhance the model’s learning process by incorporating data from various imaging modalities, potentially resulting in more precise segmentation outcomes.

## Data Availability

The dataset used in this research is publicly available. Readers are encouraged to access the data from the original source. The authors are, however, committed to promoting reproducibility and will provide the code necessary to replicate the simulations discussed in this paper upon request.

## Abbreviations

FLAIR: Fluid-Attenuated Inversion Recovery
VGG19: Visual Geometry Group 19
U-Net: U-shaped Network
TCGA: The Cancer Genome Atlas
MRI: Magnetic Resonance Imaging
TCIA: The Cancer Imaging Archive
WHO: World Health Organization
ROC: Receiver Operating Characteristic Curve
TPR: True Positive Rate
FPR: False Positive Rate
AUC: Area Under the Receiver Operating Characteristic Curve
ReLU: Rectified Linear Unit
DSC: Dice Similarity Coefficient
IOU: Intersection Over Union
BraTS: Brain Tumor Segmentation
CNNs: Convolutional Neural Networks
